# Rethinking Organisational Justice in Healthcare Through Nurses' Voices: Insights From Nancy Fraser's Theory of Justice

**DOI:** 10.1111/nup.70123

**Published:** 2026-09-27

**Authors:** Camelia López‐Deflory, Margalida Miró‐Bonet, Amélie Perron

**Affiliations:** ^1^ Department of Nursing and Physiotherapy Universitat de les Illes Balears Palma Spain; ^2^ School of Nursing, Faculty of Health Sciences University of Ottawa Ottawa Ontario Canada

**Keywords:** critical theory, health services research, healthcare management, nurses, organisational justice

## Abstract

Organisational justice has emerged as a key area of interest given the challenges of protecting the health and well‐being of nurses in healthcare organisations. Existing research shows significant conceptual and methodological limitations that must be addressed to achieve a more comprehensive and ethically grounded understanding of nurses’ needs in the healthcare system. This article explores how nurses experience their working conditions within public healthcare organisations through a theoretical lens of social justice to unveil the systemic injustices embedded within them. Adopting a critical discourse analysis approach, this study draws on 31 semi‐structured interviews with nurses working within the public healthcare sector in the Balearic Islands (Spain), informed by Nancy Fraser's feminist theory of justice. The findings illustrate that nurses conceptualised organisational justice as the condition that allows them to practice their profession in accordance with their professional ethos. This novel understanding of organisational justice underscores the economic, social, cultural and political manifestations of gendered injustices nurses face in their daily practice and that prevent them from being considered as peers in the social life of healthcare organisations. This study highlights the imperative to rethink organisational justice through a lens that accounts for the gendered nature of care and nursing work. Nancy Fraser's theory of justice emerges as a groundbreaking framework that offers novel insights to guide this transformative endeavour.

## 1 Introduction

Organisational justice refers to an employee's perceptions of their organisation's behaviours, decisions, and actions, and how these influence the employee's own attitudes and behaviours at work (Greenberg 1987). This concept encompasses three dimensions: distributive justice, which pertains to how resources should be allocated; procedural justice, which outlines the procedures that should be followed in decision‐making; and interactional justice, which is further subdivided into interpersonal justice, referring to the quality of treatment employees receive, and informational justice, which concerns the clarity and transparency of the information employees receive about organisational decision‐making processes (Greenberg and Colquitt 2005).

Research on organisational justice in healthcare organisations is primarily conducted by scholars in management and behavioural sciences, focusing on exploring and measuring the relationships between the various dimensions of organisational justice and a broad range of organisational variables. Research efforts have consistently focused on a specific study population—nurses—examining the relationship between their perceptions of distributive, procedural, and/or interactional justice and outcomes that ultimately contribute to organisational benefits, such as such as job performance and productivity (Sulander et al. 2016), organisational commitment (Chegini et al. 2019; Chen et al. 2015), organisational citizenship behaviour (Arbabisarjou et al. 2014; Chang 2014), workplace deviance (Abou Hashish 2020), job satisfaction (Chegini et al. 2019; Rodwell and Gulyas 2015; Xerri 2014), moral distress (Haghighinezhad et al. 2019; Rodwell and Gulyas 2015), work‐related stress and burnout (Ren et al. 2021), or job retention (Cao et al. 2020; Perreira et al. 2018; Sulander et al. 2016), among others.

The research conducted to date is invaluable for its ability to uncover findings that offer crucial insights into the relationship between nurses’ perceptions of justice and its far‐reaching implications for their well‐being and performance, the quality of care they deliver, and the overall sustainability of the healthcare system. However, these studies expose significant conceptual and methodological limitations that not only hinder a comprehensive understanding of the complexity of nurses’ needs within healthcare organisations but also compromise the ethical rigour that should underpin the study of organisational justice in these settings (Alzola 2011; Fortin and Fellenz 2008).

Organisational justice, as currently depicted in the literature, is framed as a predefined construct imposed on study participants. Nurses lack both individual and collective voice in defining what justice and injustice mean to them within healthcare organisations. As a result, the meaning they attribute to organisational justice remains unknown (López‐Deflory 2022). Organisational justice is also understood as a concept confined within healthcare organisations. The relationship between the factors that shape nurses’ experiences of justice and injustice and the broader systemic structure in which healthcare is delivered remains unexplored. As a result, current studies on fostering changes that go beyond mere shifts in nurses’ perceptions to transform the structural causes of organisation injustice is significantly limited (Cugueró‐Escofet and Fortin 2014; Cugueró‐Escofet and Rosanas 2017, 2013). Finally, organisational justice, although regarded as a subjective concept, is predominantly examined through quantitative research methods. An in‐depth exploration of nurses’ individual and collective experiences of justice and injustice through qualitative or mixed methods research is lacking in the literature. As a result, the how and why reeasons behind nurses’ perceptions of justice and injustice remain unaddressed and the complexity of organisational justice within healthcare settings is overly simplified (Denzin and Lincoln 2017).

Addressing the identified conceptual and methodological limitations is not only a crucial ethical responsibility but also an imperative for nursing management. It is crucial to understand organisational (in)justice by listening to and amplifying the voices of those who experience it, analysing the complex network of factors that shape it, and examining the power structures that sustain its existence. The research approach that could successfully tackle this challenge involves the application of critical and interdisciplinary theoretical frameworks on organisational justice (Alzola 2011; Fortin and Fellenz 2008; Fortin et al. 2016).

Moffa and Longo (2016) is the only one identified in the literature that utilises a social justice framework to explore nurses’ perceptions of organisational justice within healthcare organisations. In particular, these authors use the theory of well‐being dimensions developed by bioethicists (Powers and Faden 2006) to analyse workplace mistreatment as a form of injustice that not only prevents nurses from achieving their optimal well‐being in the work environment but also violates patients’ right to receive quality care. Beyond this study, no other research was identified by the researchers addressing organisational justice in the healthcare sector from a critical theoretical framework.

This article seeks to contribute to filling this gap in the literature by exploring nurses’ experiences of their working conditions in public healthcare organisations through Nancy Fraser's critical feminist lens of justice.

## 2 Background

Fraser's theory of justice conceptualises justice as the parity of participation, referring to the economic, cultural, and political conditions that ensure all members of a community can participate as peers in social interactions and in social life (Fraser 1995). Understanding this definition requires exploring how the different dimensions which allow a society to be labelled as just have evolved within the political debate.

According to Fraser (1995) justice requires two dimensions: redistribution and recognition. From the redistribution dimension, justice is understood as the equitable allocation of resources and wealth among the individuals that make up a society, that is, social equality. (In)justice is interpreted as a socio‐economic issue and, therefore, deeply rooted in the political‐economic structure of society (Fraser 1995). The solution to economic injustice would involve some type of economic restructuring that implies dedifferentiation, that is, the elimination of class or socio‐economic differences.

From the recognition dimension, justice is understood as respect for cultural differences and the rejection of the assimilation of dominant social norms and practices as a condition for the integration and equal respect of individuals within society. (In)justice is interpreted as a cultural issue and, therefore, deeply rooted in the social patterns of representation, interpretation, and communication of a given context (Fraser 2000). The solution to cultural injustice would involve some type of cultural or symbolic change involving positive differentiation—that is, the elimination of the stigma associated with status or identity differences.

Fraser contends that, contrary to traditions that analyse these dimensions in a truncated manner by asserting their analytical autonomy and opposing normative objectives, redistribution and recognition are not independent or separate dimensions. The two dimensions are intrinsically intertwined and mutually reinforcing. In practice, economic injustices are frequently compounded by a lack of cultural standing, and vice versa; consequently, any remedy that neglects one of these dimensions will prove insufficient to achieve parity of participation for all. Justice, therefore, must be understood through a perspectival dualism.

Nonetheless, to adapt her framework of justice to a globalised world, Fraser's later work undergoes a transnational shift, expanding her bidimensional model into a three‐dimensional one. While justice has traditionally been debated within the confines of the nation‐state, the author argues that the entanglement of the economic and the political also necessitates a corresponding political framework. Questioning what is just no longer suffices; there is an urgent need to interrogate who is entitled to claim justice and how the spaces of possibility for such claims are delimited (Fraser 2010).

Fraser highlighted a missing or neglected link: the political dimension and its core concept of representation. From the dimension of representation, justice addresses issues of social belonging—that is, who is included in or excluded from social life—as well as procedural matters, namely the rules or norms of debate and decision‐making that govern the possibility of participation of social life (Fraser 2010). (In)justice is interpreted as a political issue and, therefore, deeply rooted in the political structure of society, which determines the framework—the *who* and the *how*—of social interactions and social life more broadly (Fraser 2010). The remedy for political injustice would entail a restructuring of the political framework. This would involve ensuring both that internal rules of representation are inclusive and that the decision‐making framework itself does not exclude those affected by transnational structures. Only through such means can it be guaranteed that affected individuals possess the agency to struggle for redistribution and recognition within the global arena.

In conclusion, Fraser argues that there can be no justice without redistribution, recognition, and representation. The remedy to injustice does not lie in superficial solutions, but rather in dismantling the economic, cultural, and political institutional barriers that systematically prevent certain individuals from participating as peers in social life.

Fraser's theory of justice has been used in social research to address a wide range of topics related to gender equity, the welfare state, social protection, poverty, identity, and exclusion, particularly in relation to the collectivities most affected by the crisis of capitalism, such as women, the LGBTQ+ community, and ethnic minorities (Ferrarese 2015). Health research has also proven increasingly receptive to Fraser's theory of justice as a framework for addressing issues mainly related to health equity. However, this dialogue has largely remained at a macro‐structural level.

Consequently, a gap remains in the healthcare literature regarding the translation of Fraser's framework to the micro‐structural level and to delimited settings—that is, to the ‘ground’ or the point of care within healthcare organisations (Aqtam 2026). It is precisely at this level that nurses, a feminised collective of health professionals, face increasing constraints to participating in the healthcare system as peers, thereby jeopardising both the health and well‐being of this collective and the quality and safety of care provided within healthcare organisations.

In this sense, Fraser's work holds significant potential as a theoretical lens to explore the forms of (in)justice that, inherently linked to the working conditions of commodified reproductive labour, determine the relationships nurses establish with their profession, themselves, other health agents, and patients and their families. Analysing the conditions of redistribution and recognition to which nurses are subjected is key in this regard. Nevertheless, to fully mobilise Fraser's theoretical lens in this context, it is also essential to consider the frequently overlooked issue of representation or misframing (Fraser 2010). In the nursing context, this requires examining the mapping of overlapping governance layers—ranging from the macro to the micro‐level—that converge at the point of care, resulting in the obstruction of nurses’ parity of participation both within and outside healthcare organisations.

## 3 Aim

The purpose of this study is to deepen the understanding of how nurses working in healthcare organisations perceive organisational (in)justice in their daily practice. The specific research questions of this study included:

(1) How do nurses define organisational justice?

(2) What elements do nurses believe shape organisational justice?

(3) What is the relationship between this definition and these elements and the broader systemic structure in which public healthcare organisations operate?

This research will transform the current understanding of organisational justice, shifting it from a traditional view of justice as an individual experience, detached from context, to a novel, critical, ethical, and political perspective, where justice is seen as an experience rooted in the economic, cultural, and political structures of the society in which healthcare organisations operate. By shifting the analysis and interpretation of nurses’ perceptions of organisational justice, this study aims to generate new knowledge that can inform nursing management. The insights gained will help address and remediate the organisational (in)justices that nurses face in their daily practice, fostering holistic solutions that contribute to positive outcomes for nurses, patients, and healthcare organisations.

## 4 Methods

### 4.1 Study Design

This study adopts a qualitative, critical discourse analysis (CDA) research design. CDA is a form of social research focused on the study of language as a means of uncovering the ways in which discourses construct and mediate the naturalised socio‐cultural and political realities of a given historical and social context (Fairclough 1995). This design is particularly suited to gain a deep understanding of how nurses perceive justice within their work environment and, consequently, explore how injustice is constructed, sustained, and/or challenged within public healthcare organisations. Furthermore, this study was explicitly driven and guided by theory. The chosen theoretical framework was Nancy Fraser's theory of justice, which was introduced from the outset and remained central throughout all phases of the research (Grant and Osanloo 2014).

### 4.2 Reflexivity and Positionality

The research team positioned itself within a critical qualitative paradigm. The study was led by the first author—a nurse‐academic and qualitative researcher attuned to structural injustices in healthcare organisations and the advocacy of nurses’ political agency. The co‐authors are similarly nurse‐academics who employ critical social theories to interrogate institutional factors detrimental to nursing practice. This shared critical positionality and ongoing collective reflexivity directly shaped the study's design, analytic process, and interpretation, ensuring a sustained critical engagement with structural inequalities in healthcare settings.

### 4.3 Study Context

This study was conducted in the Balearic Islands, Spain. In this context, nursing practice is shaped by multiple layers of governance; while many are shared with other regions, others remain specific to this territory. The critical nature of this study necessitates rendering these layers visible to facilitate their transferability to other research contexts.

Spanish national health legislation recognises nurses as degree‐qualified healthcare professionals who possess professional autonomy and responsibility regarding health promotion, prevention, care, and rehabilitation (Ley 44/2003). In Spain, this basic regulatory framework for nursing practice is embedded within a public healthcare model characterised by decentralised management, which is administered by regional governments (Ley 1986). Legislation promoted by regional governments intersects with hospital‐level management policies—which govern shift organisation, staffing allocation, supervisory styles, and channels for participatory decision‐making—and, in turn, with professional associations that determine standards of practice and professional norms. These governance layers mold both the professional life and the status of nurses within and outside Spanish healthcare organisations.

In the Balearic Islands, these layers intertwine to construct a nursing reality characterised by work environments where health austerity measures translate into high workloads and elevated nurse‐to‐patient ratios—among the highest in the European context, in line with national averages (Ministerio de Sanidad de España 2025). Furthermore, patriarchal practices impact interprofessional relationships, which remain markedly hierarchical as a result of a historical legacy of National Catholicism (Miró et al. 2012). Finally, non‐inclusive organisational dynamics illustrate a tension between the scarce voice of nurses in clinical and non‐clinical decision‐making spheres and the significant representation of nurses in managerial and political positions compared to other Spanish territories (Santillan‐Garcia et al. 2020).

### 4.4 Participants

The study participants included 31 nurses holding various positions within the public healthcare organisations of the Balearic Islands, encompassing primary care, hospital care, and long‐term care settings. These roles comprised nurses in clinical practice, middle nurse managers, nurse managers, nurses in political and/or professional association roles, union representatives, and nurses in mixed roles (combining clinical and management responsibilities). The inclusion of diverse nursing profiles aimed to capture the nuances in how organisational injustice is experienced across different levels and functions within healthcare organisations.

Participants were selected using intentional sampling. The sample was stratified by sex/gender to align with the demographic distribution of the target population. This stratification is central to the study's framework given the historically gendered structure of the nursing profession, where gender operates as a primary axis shaping organisational justice within a predominantly female workforce. Furthermore, this strategy was contextually tailored to the local healthcare setting, where sex/gender—intersected with professional hierarchy—constitutes the most salient structural determinant of institutional inequality. Participants were required to meet three inclusion criteria: (a) be working at a public healthcare organisation at the time of data collection; (b) have at least 6 months of work experience at a public healthcare organisation; (c) voluntarily agree to participate in the study and sign the informed consent form.

### 4.5 Data Collection

This study was conducted through semi‐structured, in‐depth, face‐to‐face interviews. The interviews were carried out by the lead researcher, a woman, nurse, and experienced qualitative researcher, who had prior training in this data collection technique. An open‐ended interview guide was developed to explore the participating nurses’ perceptions of (in)justice as experienced in their daily practice. The guide was piloted with two participants prior to implementation. No modifications were necessary, as participants did not report any difficulty understanding the questions and felt that the guide allowed them to express themselves freely. Some of the questions included in the guide were: 'What does being a nurse mean to you?', 'Do you think the way you currently practice the profession differs from how you would like to practice it? Why?', and 'What are the main challenges you face as a nurse, and what do you think are the main challenges the nursing profession as a whole is facing?'

Interviews were conducted in the preferred language (Spanish or Catalan), date, time, and location selected by each participant between 2021 and 2022. All interviews were audio‐recorded with participants’ prior consent, transcribed immediately afterwards, and used for subsequent analysis. The interviews lasted between 45 min and 2 h, with an average duration of 90 min. A sociodemographic questionnaire was also administered to collect data such as sex, years of experience, and current field of practice. Additionally, the lead researcher kept a field diary in which theoretical, methodological, and descriptive notes were recorded. Although the fieldnotes were not systematically analysed as a separate dataset, they facilitated contextual understanding of the interview data and were used to support reflexive interpretation, thereby enriching the data analysis process.

### 4.6 Data Analysis

A critical discourse analysis was conducted on the data following Norman Fairclough (1995) framework, which conceptualises discourse not merely as language use, but as a form of social practice inherently bound up with power relations, ideology, and institutional structures—analysed through text, discursive practice, and social practice. Data analysis was carried out concurrently with data collection. Audio‐recorded material was transcribed verbatim and analysed in five phases. In the first phase—familiarisation—transcripts were repeatedly read to identify general key ideas. In the second phase—identification—salient discursive patterns were identified across data. In the third phase—indexing—data were coded using both inductive and deductive strategies, resulting in the development of codes that captured both textual elements and underlying power dynamics. In the fourth phase—connection—codes were grouped into subcategories, and subcategories into broader categories, based on shared meanings. In the fifth and final phase—interpretation—the codes, subcategories, and categories were examined in relation to existing literature and Nancy Fraser's justice theoretical framework to address the research questions guiding the study.

To illustrate the findings, representative verbatim excerpts used in this manuscript were translated from Spanish/Catalan into English through a collaborative process between the principal investigator and a professional translator, minimising semantic distortion and preserving the original nuances of participants’ accounts.

### 4.7 Rigour

The rigour strategies used to ensure the reliability and credibility of the study followed those recommended by Morse (2015). These strategies included: (1) validity, ensured through member‐checking techniques with participants, systematic transcription of interviews, inclusion of negative cases in the analysis, and exemplification of codes using verbatim excerpts; (2) reliability, maintained through a detailed account of the study's methodological considerations and the principal investigator's ongoing reflexivity throughout the research process; and (3) generalisability, supported by a comprehensive description of the study context and participant profiles, broad inclusion criteria, incorporation of extreme cases, data analysis until saturation, and adherence to a specific theoretical framework to prevent artificial coherence in the findings.

### 4.8 Ethical Considerations

This study was approved by the Research Ethics Committee of the Balearic Islands (Ref. 4013/19). Participation in the study was voluntary. All participants were provided with comprehensive written and verbal information regarding the objectives, procedures, and nature of the study and had to sign a written informed consent form prior to enrolment. The researcher conducting the interviews sought to ensure a safe environment that encouraged participants to openly share their thoughts and experiences. All identifiable information was anonymised, all participants were assigned pseudonyms, and strict confidentiality was maintained throughout the research process.

## 5 Findings

**FIGURE 1:**
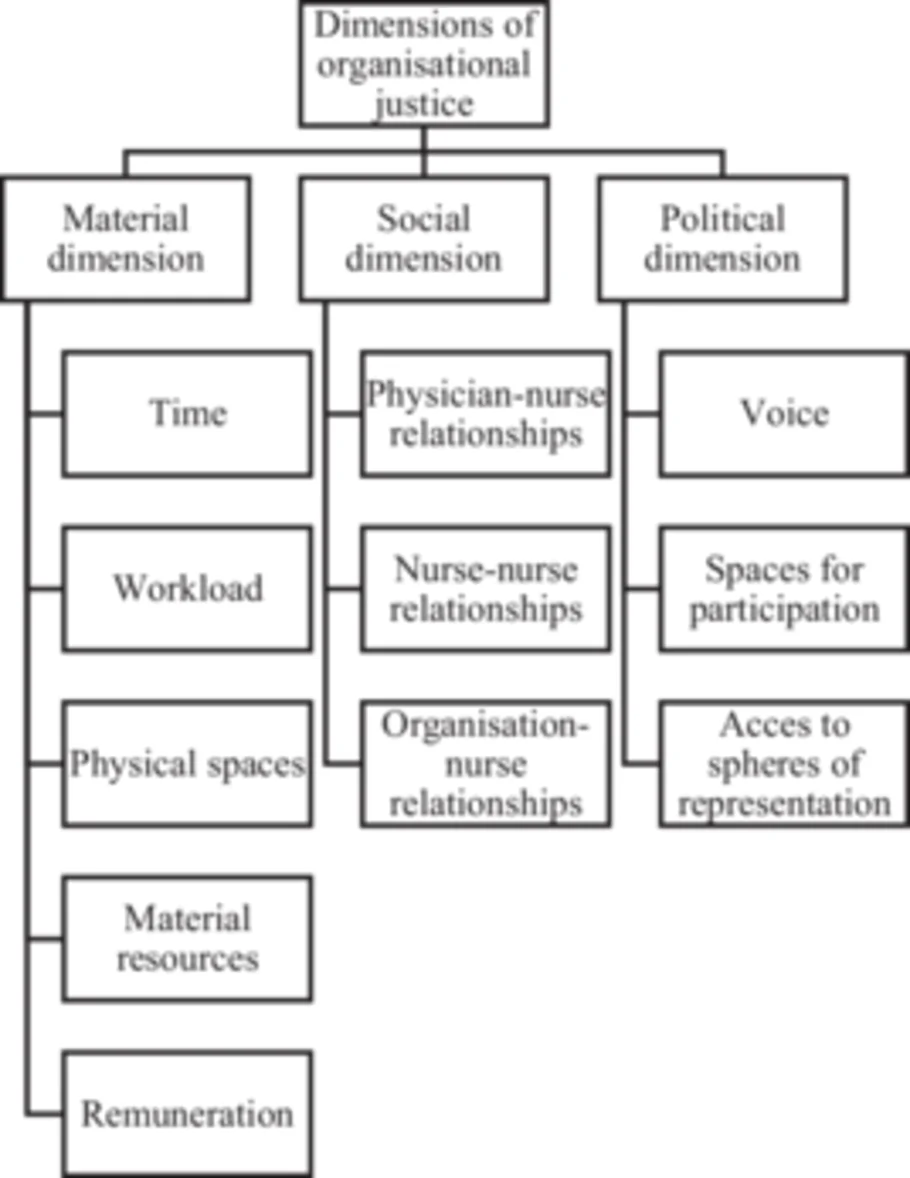
Dimensions of organisational justice from nurses’ perspectives.

The participating nurses developed their own conceptualisation of organisational justice by analysing the factors they perceived as limiting the full expression of their professional roles, which in turn restricted their ability to provide optimal care to patients. The critical discourse analysis conducted on the collected data resulted in three categories. Each of these categories corresponded to one of the three dimensions of elements described by the participants. Figure 1 illustrates the three identified dimensions and the specific elements within each. The aim of this section is not to provide a detailed account of the participants’ reflections, but rather to present a mapping of the elements that, from their standpoint, shape organisational justice.

### 5.1 Material Dimension

Nurses initially referred to the components constitutive of the material structure of healthcare organisations. These components aligned with the traditional conceptualisation of working conditions, encompassing dimensions such as time, workload, physical spaces, material resources, and remuneration. Participants examined these elements through the lens of social comparison, primarily drawing parallels with other healthcare professionals, particularly physicians. Such comparative discourses allowed them to discern professional disparities and fostered a critical awareness of their own inequality within healthcare institutions. Furthermore, this comparative process enabled the articulation of specific grievances regarding their labour conditions—both in terms of parity concerning time, workload, spaces, and resources, and in terms of the systemic revaluation regarding their remuneration.

Nurses framed their working hours as rigid, fast‐paced, and scarce. They expressed feeling confined within a chrono‐practice, deploying metaphors of working in a factory‐like environment to illustrate how strict schedules dictated the routine execution of mechanical, sequential tasks. Additionally, through these textual accounts, they conveyed frustration over the lack of time to address their own needs, reflect on their professional practice, or engage in non‐clinical activities such as teaching, research, or advocating for their profession.

*The hospital feels like a factory to me, and I think this is a widespread feeling*. (…) *Our work is becoming dehumanised because you have contact with the patient, but you really end up feeling like a robot ·* Julia [Nurse in clinical practice · Female · Hospital Care 1]

*The unit is a factory*. (…) *You arrive at 8:00 AM and check everything. You check the schedules. You check the treatments. You prepare the machines. By 8:25 AM, everything has to be ready because we need to welcome the patients. You have 7 min to connect the patient to the machine and the same amount of time to disconnect them*. (…) *On a good day, you get 35 min for lunch; on a bad day, 15–20 min. You eat and go back to the ward. Then you start the afternoon shift and do exactly the same. That's my shift ·* Martín [Nurse in clinical practice · Male · Hospital Care 2]

*We need a lot of personal self‐care, but we don't have the time or space to do it ·* Javier [Nurse in clinical practice · Male · Primary Care 5]

*There's an incredible lack of time. I'd love to do some research, but this morning I didn't sit down for even a second ·* Ana [Nurse in clinical practice · Female · Hospital Care 5]

Nurses linked their reflections on working hours to their workload, constructing a narrative of being systemically overwhelmed. In their accounts, they attributed this workload overload to external factors—such as high nurse‐patient ratios, the need to adapt to technological innovations, the intrusion of bureaucracy, and the inherent characteristics of the profession—as well as internal factors, such as their commitment to the quality of care provided and the achievement of organisational goals.

*I don't think we have a good allocation and distribution of resources because it's based on ratios, like ‘so many nurses for so many patients’*. (…) *And it doesn't take into account the intensity of care, the levels of dependency, or the profile of the patients ·* Daniel [Nurse manager · Male · Primary Care 2]

*When I started working, there was a gadget here and there around the hospital, just in case it was needed* (…)*. Now, there are gadgets everywhere, they've invaded the profession. They're in every corner* (…) *And that's just more workload for the nurse ·* Martín [Nurse in clinical practice · Male · Hospital Care 2]

*Administrative tasks shouldn't be there, but they are* (…) *I think it's important to consider that if there's more and more paperwork, in the end, you need more nurses or you need to give nurses time to deal with it ·* Lara [Nurse in clinical practice · Female · Hospital Care 7]

*As a nurse, you have to bear heavy workloads*. (…) *Someone working in an office probably won't experience those peaks of frantic activity that certain professions, like ours, have, right? And there's not much you can do about it, it's just part of the job · Paula* [Nurse manager · Female · Hospital Care 3]

*I have a lot of work, much more than I might actually be able to do because* (…) *it's really hard to see needs and not respond to them. This creates a significant workload for me*. (…) *The more you do, the more work you get, you know? And sometimes you think, ‘I'm overloading myself…'* · Elena [Nurse in clinical practice · Female · Primary Care 3]

Nurses also analysed the physical spaces in which they worked, characterising them through a lexicon of constraint as small, uncomfortable, and impractical. They also reported a lack of spaces for exchanging clinical information, developing care plans, holding intra‐professional meetings, and meeting their own needs. The design of the workspaces—which discursively and structurally prioritises the needs of physicians rather than those of patients—and the lack of nurse representation in decisions concerning these issues were identified as key institutional mechanisms reproducing this condition.

*The space is very small. You can't move properly to perform the techniques, and if something unexpected happens, it's difficult to do the job properly* · Julia [Nurse in clinical practice · Female · Hospital Care 1]

*We don't have a room to meet in*. (…) *We have to sit on the floor in the surgery reception area. There are no chairs. No tables. You just sit on the floor. That's how we have our meetings… ·* Clara [Nurse in clinical practice · Female · Hospital Care 6]

*We don't have spaces to eat. We have a small table where four or five of us can fit. There's no staff bathroom; we share one with the patients ·* Martín [Nurse in clinical practice · Male · Hospital Care 2]

*Organisations aren't designed with the needs of users in mind, but rather with the needs of professionals in mind. Of certain professionals! Physicians ·* Daniel [Nurse manager · Male · Primary Care 1]

*Nobody, ever, asked me how the service should be. I never sat down with the architect. Never! Never! The hospital is designed by doctors. That's how clear it is! ·* Paula [Nurse manager · Female · Hospital Care 3]

Nurses shared their concerns regarding the material resources available to carry out their work, constructing these resources as either quantitatively insufficient or qualitatively inadequate. In challenging dominant managerial discourses, participants identified the culture of cost‐saving, the bureaucratisation of procurement processes, and the exclusion of nurses from decision‐making as the main social practices driving resource scarcity.

*There's a lack of material. You don't have everything you need at all times, and you have to improvise ·* Pablo [Nurse in clinical practice · Male · Primary Care 1]

*Well, there are enough, I believe, yes. But when it comes to quality… definitely not* (laughs) *·* Julia [Nurse in clinical practice · Female · Hospital Care 1]

*Everything is very tightly rationed ·* Cecilia [Nurse in clinical practice · Female · Primary Care 4]

*It's very complicated due to bureaucracy*. (…) *We have to make all requests through orders that go to the hospital, someone reads them, and sends you an email…* (…) *Just to give you an idea, this morning we received items that were requested in last year's needs plan ·* Silvia [Middle nurse manager · Female · Hospital Care 1]

*The purchasing manager is the one who decides what resources we have and what we don't ·* Julia [Nurse in clinical practice · Female · Hospital Care 1]

Finally, the nurses articulated their views on their remuneration. While some participants expressed satisfaction with their salary, the majority discursively constructed their pay as a symbolic reflection of institutional devaluation, asserting that it was not aligned with the responsibility they assumed or the inherent demands of nursing work.

*I believe we are reasonably well paid. Could we be paid better? Yes… The issue is that we carry a very high level of responsibility, and overall, this responsibility is not sufficiently recognised or compensated* · Daniel [Nurse manager · Male · Primary Care 1]

*I don't feel adequately compensated*. (…) *The family expenses, socialising with friends, working on holidays, at night, on Sundays… everything you're exposed to: the risks associated with isolated patients, biological hazards…* (…) *And personally, you also witness many things that leave a mark on you!* (…) *What can I say? Every penny is well earned! And I believe we should be paid more for everything that comes with it* · Lara [Nurse in clinical practice · Female · Hospital Care 7].

### 5.2 Social Dimension

Nurses constructed the social structure of healthcare organisations through a sustained critique of interprofessional dynamics—primarily with physicians and fellow nurses—and their relationships with institutional frameworks. Rather than presenting neutral accounts of workplace relationships, participants articulated a collective claim for recognition. This discursive stance was grounded in highlighting the distinct value of nursing contributions to clinical care processes and challenging the systemic undervaluation of their work within the healthcare system.

Participants framed their relationships with physicians through narratives of distance, territoriality, and hierarchy. They described how dominant discourses position physicians as authoritative team leaders, fostering instrumental behaviours towards non‐medical staff. In their accounts, participants conveyed that their contributions were routinely undervalued or inadequately acknowledged by physicians, and that they were often subjected to disrespectful treatment.

*The relationship between physicians and nurses is very cordial, but we are separate. I mean, it's medicine and nursing, and ‘they are physicians’ and ‘we are nurses'* ·Lucía [Middle nurse manager · Female · Hospital Care 3]

*Physicians live in their cloud of being great professionals… They love to make it seem like they are the ones in charge and that you're just the nurse* · Diego [Nurse in clinical practice · Male · Hospital Care 3]

*I think there are a lot of barriers between us because physicians think we want to take away the privileged position they've always had ·* Claudia [Nurse in a political or professional association position · Female · 2]

*There are physicians who still think nurses are in the system to make their lives easier ·* Elena [Nurse in clinical practice · Female · Primary Care 3]

*Physicians don't really understand what a nurse is or what their job entails ·* Nadia [Nurse in clinical practice · Female · Primary Care 2]

Nurses also interrogated intra‐professional relationships, examining interactions among staff nurses as well as between staff nurses and middle nurse managers. Reflections regarding peer‐to‐peer relationships revealed interpersonal tensions marked by a perceived lack of solidarity and recognition. Participants constructed these strained dynamics as a consequence of fragmented understandings regarding the core ontological and ethical foundations of the nursing profession.

*My relationships with my colleagues are good, but the issues we have stem from the fact that we differ in how we understand the profession ·* Martín [Nurse in clinical practice · Male · Hospital Care 2]

*Yes… It's mostly about that: the different views we have about who we are, what our work is, and what our purpose should be ·* Nadia [Nurse in clinical practice · Female · Primary Care 2]

*I usually get along with everyone, but there are nurses who are incredibly negligent, who just don't care. They come in to pass the time, do only what's strictly necessary, the bare minimum expected of them, and they just have no desire to do the job ·* Olivia [Nurse in mixed role · Female · 2]

*I get along with my colleagues because I have to work with them, but I don't feel recognised. I don't feel respected by them ·* Clara [Nurse · in clinical practice Female · Hospital Care 6]

In constructing their accounts of relationships with middle nurse managers, participants highlighted hierarchical dynamics and feelings of vulnerability. Nurses in clinical practice framed middle management as an intermediate institutional layer primarily aligned with organisations’ interests and operation rather than staff wellbeing, reducing managerial practice to administrative oversight and shift management.

*The middle nurse manager is above us and stands between the clinical level and the management level ·* Valeria [Nurse in clinical practice · Female · Primary Care 6]

*Our middle nurse manager is more like an office‐based supervisor. Her only role seems to be covering work shifts ·* Ingrid [Nurse in clinical practice · Female · Hospital Care 4]

*The supervisor doesn't look after us. She's more concerned with pleasing those higher up, the management… ·* Julia [Nurse in clinical practice · Female · Hospital Care 1]

Nurses also reflected on their relationships with healthcare organisations as whole entities. They constructed institutions as indifferent structures that unrecognise and instrumentalise nurses to meet organisational needs. They reported feeling treated as resources that could be moved from one place to another, regardless of their expertise, the nature of the tasks to be carried out, or their individual situation and personal needs.

*Healthcare organisations are fully aware of the conditions we're working under here, but they simply don't care ·* Lara [Nurse in clinical practice · Female · Hospital Care 7]

*We're just disposable resources ·* Olivia [Nurse in mixed role · Female · 2]

*They decide where to place you based on the available staff*. (…) *My knowledge, experience, or personal situation aren't taken into account at all ·* Clara [Nurse in clinical practice · Female · Hospital Care 6]

*They take advantage of you a lot. They call you saying, ‘Would you be interested in working at such‐and‐such place today?’ They don't think about the fact that you have a life, and when you say no, they get upset ·* Valeria [Nurse in clinical practice · Female · Primary Care 6]

Finally, nurses addressed external governance structures, such as trade unions and professional associations. In their accounts, participants expressed widespread criticism towards these organisations, discursively constructing them as disengaged entities in which they lack trust, as they found no evidence that these bodies truly advocate for their well‐being within healthcare institutions.

*In terms of trade unions, I think it's a shame*. *I don't believe in them because they don't represent me. The union is just a figure that's there and does little for the profession* · Nadia [Nurse in clinical practice · Female · Primary Care 2]

*If you ask me, 'Do you know what the College has done lately?' I don't know! The only thing I know is that I once went on the college's website and only saw holistic courses*, *and that's it!* · Diego [Nurse · in clinical practice Male · Hospital Care 3]

### 5.3 Political Dimension

Nurses finally addressed the components constitutive of the political structure of healthcare organisations. Their reflections centred on the expression of their professional voices, their involvement in decision‐making processes, and their access to spheres of professional and political representation. Through these accounts, participants mapped an institutional framework that, driven by a subordinating logic, systematically marginalises them based on their professional identity. In response to this perceived disenfranchisement, participants articulated a demand for political legitimacy—seeking to position the distinctiveness of nursing knowledge as binding both within clinical teams and across broader governance arenas.

Nurses framed their voices as limited, unheard, and ineffective when compared to toher healthcare disciplines. They revealed that their clinical insights were relegated to to immediate patient care and solicited primarily when supporting the execution of medical or institutional agendas, rather than serving as an autonomous platform for professional advocacy. They were heard, but only listened to by patients to relay information from other professionals, by physicians when supporting the execution of their own professional role, and by healthcare organisations when contributing to organisational goals. Through their accounts, nurses highlighted how their voices lack the political authority required to shape healthcare and organisational processes.

*Many patients who leave the physician's consultation come to me and ask: ‘I didn't quite understand what the doctor told me, can you explain it to me?’ ·* Lara [Nurse in clinical practice · Female · Hospital Care 7]

*Sometimes, physicians don't listen to you the first time, there needs to be a second, and sometimes even a third time ·* Cecilia [Nurse in clinical practice · Female · Primary Care 4]

*The organisation doesn't listen to you until it's interested, meaning, it uses you and then discards you…·* Martín [Nurse in clinical practice · Male · Hospital Care 2]

*Our words fall on deaf ears ·* Nadia [Nurse in clinical practice · Female · Primary Care 2]

Nurses linked their limited professional voice to the constrained participatory spaces available within healthcare institutions. Clinical decision‐making was constructed not as a collaborative or democratic arena, but as a site of medical dominance. Participants framed this communicative asymmetry as a source of moral distress for nurses and an ethical risk for patient care, illustrating how non‐democratic decision‐making excludes nursing perspectives from influencing clinical governance.

*The physician is the one who takes the lead in decision‐making ·* Diego [Nurse in clinical practice · Male · Hospital Care 3]

*It's supposed to be a joint decision‐making process, but in the end, the physician's opinion always prevails… Nurses are consulted, or rather, informed* · Lara [Nurse in clinical practice · Female · Hospital Care 7]

*Nurses experience moral distress when physicians make decisions they don't agree with, and they have no opportunity to discuss or influence them. I think this is also an injustice towards the patient, as it prevents a particular clinical judgement from being open to other perspectives ·* Gemma [Nurse manager · Female · Hospital Care 1]

Silences in nurses’ accounts exposed the absence of systematic, institutionalised spaces for organisational decision‐making. Through counter‐narratives of structural exclusion, participants critiqued top‐down administrative hierarchies, highlighting the paradox of executing operational changes without having a binding voice in their design.

*I believe the hierarchy is inverted because we depend heavily on the directors, sub‐directors, and the entire chain of command… We're left until the very end. I think it should be the other way around. We are the ones who have to implement the structural decisions that are made. We should be the ones to know whether these changes can be positive or negative, but ask us! There is no dialogue or communication* (…) *We can't make decisions. ·* Ingrid [Nurse in clinical practice · Female · Hospital Care 4]

Finally, nurses examined their access to spheres of professional representation—such as in middle management and healthcare executive roles—, and political leadership. Participants constructed these opportunities through metaphors of severe structural containment—characterising the barrier to leadership roles as a 'cement ceiling' sustained by external medical resistance as well as internalised professional hesitation.

*The glass ceiling for nurses is made of cement ·* Claudia [Nurse in political or professional association role · Female · 2]

*So far, there has been no nurse coordinator in the centre* (…) *and this has been quite a significant shock*. (…) *It seems that part of the medical establishment thinks that this is an offence to their superiority* (…) *and that we cannot contribute all that we have to offer in this position* · Emma [Nurse manager · Female · Primary Care 2]

*I think it is us nurses who are not accepting these roles* · Olivia [Nurse in mixed role · Female · 2]

*'I believe that, first and foremost, we need to believe more in ourselves because if we believe in our potential, we can achieve being more present everywhere'* · Julia [Nurse in clinical practice · Female · Hospital Care 1]

## 6 Discussion

This study aimed to deepen the understanding of how nurses working in healthcare organisations experience organisational (in)justice in their daily practice. Nurse participants acted as a lens through which the material, social, and political dimensions of healthcare institutions were observed, leading to a more comprehensive framework of organisational justice—one that more accurately reflects the nature of the environments in which care is provided.

Nurses articulated the foundations of a notion of organisational justice that challenges the dominant frameworks currently prevailing in the literature (Rupp et al. 2017). They moved beyond the longstanding paradigm that conceptualises organisational justice as comprising distributive, procedural, and interactional dimensions (Cropanzano et al. 2015). Instead, they advanced a new, more critically informed paradigm—one that captures the complex interplay of material, social, and political needs embedded in nursing work and care work more broadly.

Participants explored the meaning of justice through their experiences of injustice, in relation to three dimensions they themselves constructed to define the institutional order of healthcare organisations. As Moore (1978) suggests, injustice precedes justice. Nurses conceptualised injustice as the experience of being unable to care for patients in the way they wished, and justice as the experience of being able to practise their profession in a manner that resonates with their professional ethos.

First, participants shared their experiences regarding what they constructed as the material structure of healthcare organisations. They critically analysed the availability of time to carry out their tasks, the workload burdens they were expected to bear, the physical spaces and material resources at their disposal, and their level of remuneration. The findings of this study are consistent with previous research that explores nurses’ perspectives on working conditions that limit their ability to provide high‐quality patient care (Govasli and Solvoll 2020; Selberg 2013; Vinckx et al. 2018). However, the participants in this study approached these elements from a distinct standpoint, viewing them through a justice‐oriented lens that transcends the organisational orientations commonly found in the literature. They understood these elements as being inequitably distributed within healthcare organisations, attributing the causes of this maldistribution to a broader structural rift in the capitalist economic system, which subordinates care to market imperatives (Fraser 1995; Fraser 2022).

Nurses’ narratives underscored the need to rethink the allocation of time, workload, physical spaces and resources within healthcare organisations, as well as the revaluation professionals remuneration, as foundational strategies for sustaining justice within the healthcare system itself.

Secondly, participants shared their experiences concerning the social dimension of healthcare organisations, encompassing their relationships with physicians, fellow nurses, as well as their interactions with the institutions in which they work and through which they are professionally organised. The findings of this study aligned with results from previous research exploring nurses’ perspectives on interprofessional collaboration (Levesque et al. 2022), nurse–physician collaboration (Labrague 2025), horizontal violence among nurses (Blackstock et al. 2018), and the treatment received from healthcare institutions (Valizadeh et al. 2018). However, once again, participants addressed these issues from a perspective that is seldom found in the literature. They integrated this social dimension into their conceptualisation of working conditions, approaching it as a matter of justice centred on professional recognition.

While participants initially treated recognition as an interpersonal issue, the analysis of their narratives delineated the presence of underlying harmful cultural patterns of social representation (Fraser and Honneth 2003). In this sense, nurses’ reflections thus illuminated how sociocultural patterns—particularly those that render care work invisible or devalued as a feminine responsibility, and that impose expectations of selflessness and sacrifice on women in caring roles—profoundly shape nurses’ everyday interpersonal encounters at work (Fraser 2022).

Nurses’ narratives highlighted the urgent need to foster cultural change within healthcare organisations from a gender‐aware perspective, so that workplace relationships are grounded in principles of equity, respect, and autonomy, thereby enhancing both nurses’ wellbeing and the quality of patient care.

Thirdly, participants shared their experiences concerning the political dimension of healthcare organisations, addressing the expression of their professional voice, their involvement in decision‐making processes, and their access to professional and political representation spheres. The findings of this study were consistent with previous research exploring nurses’ perspectives on their professional autonomy (Mrayyan et al. 2024), their participation in clinical (Arends et al. 2022) and non‐clinical decision‐making processes (Kanninen et al. 2022; de Kok et al. 2023), as well as their involvement in various political participation bodies (Han and Kim 2024; Wilson et al. 2022). Once again, participants approached these political elements from a standpoint that remains largely overlooked in the literature. They framed them as core working conditions and as matters of justice centred on professional representation (Fraser 2010).

Nurses’ narratives allowed for the conceptualisation of representation as ‘framing issues’—that is as matters concerning who is included and/or excluded from the social life of healthcare organisations, and how formal and informal mechanisms shape the extent to which professionals are able to participate as peers both within and beyond these settings (Fraser 2008). The results indicated that nurses’ individual and collective voices are frequently excluded from deliberative processes; even when included, they often remain subject to informal norms that prevent them from being recognised as interlocutors with genuine influential power.

Their narratives underscored the need to reconsider traditional models of governance within healthcare organisations in order to secure an autonomous voice for nurses in the health system, and to prevent the far‐reaching individual, collective, and societal consequences that arise when nurses’ voices are marginalised in healthcare delivery (Drenkard 2015; Sundean et al. 2017; Sundean and Polifroni 2016).

The findings of this study have, in sum, contributed to the construction of a renewed emic and etic notion of organisational justice. This notion simultaneously considers how nurses themselves interpret, experience, and make sense of (in)justice in their working environments, and how e and how those experiences can be contextualised. Fraser's framework emerges as a normative theoretical lens capable of supporting the reconceptualisation of justice within healthcare organisations, as perceived and enacted by nurses (Cugueró‐Escofet and Fortin 2014).

However, far from merely mapping the systemic injustices affecting nurses, Fraser's framework proves instrumental in illuminating how these inequities operate within a complex web of overlapping governance layers that subsist beneath and beyond the formal organisational chart, effectively constraining nurses’ agency at the point of care. The analysis of participants’ narratives—particularly their reflections on the aetiology of injustice—elucidate how nursing practice is embedded within a material, social, and political structure. Its roots are nourished by the convergence of macro‐legal and financial layers, where national and transnational health policies dictate healthcare budgets and priorities; a socio‐cultural layer, where gendered identity norms influence professional roles; an ethical layer that establishes professional regulatory codes; and administrative and clinical protocols which guide daily nursing interventions. These competing forces position nurses as non‐peers both within and beyond healthcare organisations, consequently fracturing the care structures as a whole.

The struggle for justice in nursing is thus framed as being about more than just securing greater resources (redistribution), improved status (recognition), or an autonomous and influential voice (representation). It entails simultaneously navigating the incommensurability between nurses’ demands for equality and the recognition of their professional difference. Ultimately, it requires reframing and transforming the very scale at which decisions are made—contesting who is included, how care is valued, and from which locus of power health governance is exercised.

## 7 Limitations

The main limitations of this research relate to some methodological considerations. The sample of participants in this study may not have included nurses whose unfavourable organisational experiences made them reluctant to speak about their work environment. The measures taken to ensure participant safety during the research, including all ethical considerations, may not have been sufficient to alleviate these concerns among all potential participants. The sample of participants may also have failed to capture the intersectionality of certain social determinants, such as nurses’ ethnicity. While this dimension may not be particularly salient in the context in which the study was conducted, it could hold significant relevance in other settings. Future research is needed to explore the intersections between professional discipline, gender, and ethnicity in shaping experiences of organisational justice. Finally, although the findings of this study are not generalisable to other contexts, the use of a normative theoretical lens in the interpretation of the data strengthens their transferability to settings with similar characteristics, thereby enhancing their applicability and relevance in comparable contexts.

### 7.1 Implications

This study holds immediate value for healthcare management practices and policies. Rethinking organisational justice within healthcare settings through critical theoretical frameworks—specifically through a gender‐sensitive lens such as Fraser's theory of justice—represents a groundbreaking shift in how we understand the factors shaping organisational justice in these environments, and consequently, in how strategies should be approached to foster fairer, more democratic, and safer care environments for both healthcare professionals and patients. The findings of this study demonstrate the critical value of providing nurses with opportunities to openly discuss their perspectives on justice within healthcare organisations. This enables a deeper understanding not only of the fundamental needs of caregivers, but also of what is systematically missing—economically, socially, and politically—in current healthcare systems.

Creating formal, democratic spaces both within and outside healthcare organisations dedicated to addressing these issues, where nurses are guaranteed a meaningful seat at the table, constitutes a vital public good. Nurses’ perspectives on their daily professional practice must be heard, and each form of injustice they encounter must be actively addressed if meaningful institutional transformation is to be achieved. Targeted measures to remedy economic injustices—traditionally associated with conventional views on nursing working conditions, such as policies aimed at redistributing working time and workloads, enhancing access to material resources, or improving remuneration—are essential, yet represent only a fraction of what is required.

Equally imperative are measures designed to redress social injustices by flattening daily interprofessional hierarchies within healthcare organisations and transforming, in the long term, the sociocultural patterns that systematically devalue care and those who provide it. Likewise, political injustices must be prioritised through the formal inclusion and active listening of nurses’ voices in decision‐making spaces that affect both their practice and the population. Failing to adopt such a multidimensional framework of organisational justice will, at best, merely shift nurses’ perceptions while leaving the underlying foundations of structural injustice intact, ultimately continuing to jeopardise the well‐being of nurses and the quality and safety of patient care.

## 8 Conclusions

This study aimed to deepen the understanding of nurses’ experiences of organisational (in)justice in their daily practice within healthcare organisations. The participating nurses created a space for reflection on their individual and collective status within healthcare organisations, the various governance layers to which they are subjected, and the inherent tensions in their claims for justice to be recognised as peers in social life, both within and beyond the healthcare system. Fraser's theory of justice has been presented as a powerful analytical tool for understanding these issues. It also serves as a robust normative tool for guiding managerial decisions to develop and implement ethically grounded policies that support nurses’ well‐being and ensure their full and equitable participation as peers within the healthcare system.

## Funding

The authors have nothing to report.

## Ethics Statement

This study was approved by the Research Ethics Committee of the Balearic Islands (Ref. 4013/19).

## Conflicts of Interest

The authors declare no conflicts of interest.

## Data Availability

The data that support the findings of this study are available on request from the corresponding author. The data are not publicly available due to privacy or ethical restrictions.
